# Real-World Long-Term Experience on Endoxifen in Bipolar Disorder with Psychotic Symptoms

**DOI:** 10.1155/2022/3684181

**Published:** 2022-07-02

**Authors:** Vikas Thanvi

**Affiliations:** Dr. Thanvi's Neuropsychiatry and Psychotherapy Centre, Jaipur, Rajasthan 302019, India

## Abstract

Evidence suggests that inhibition of protein kinase C (PKC) signalling may have a contributing role in the treatment of bipolar affective disorder (BPAD). Endoxifen, an active metabolite of tamoxifen, is a potent direct PKC inhibitor. This report presents a severe case of a BPAD patient with a baseline Young Mania Rating Scale (YMRS) score of 49, associated family history and addiction to psychostimulants, with no improvement by the first and second-generation antipsychotics. Treatment with endoxifen 8 mg once a day showed improvement in manic symptoms with a YMRS score of 4 and a reduction in the use of psychostimulants as well as other antipsychotic concomitant medications. No adverse effects were noted up to 8-month follow-up. Long-term treatment with endoxifen is safe and effective in severe BPAD.

## 1. Introduction

Bipolar affective disorder (BPAD), also known as manic depressive illness, is a common mental disorder characterized by episodes of mania/hypomania with alternate/concomitant episodes of depression. Population prevalence of BPAD in lifetime is approximately 0.5–1.5% and, if untreated, is associated with impulsive activity and risk of suicide [1, 2]. Evidence based guidelines recommend the use of second-generation antipsychotics, lithium, and valproate in adults with acute mania as the first-line monotherapy [3–5]. Protein Kinase C (PKC) appears to play a pivotal role in the pathogenesis of BPAD supported by the effectiveness of PKC inhibitors, such as tamoxifen and its metabolite endoxifen, in treating manic symptoms [6]. Endoxifen is a promising novel antimanic and mood-stabilizing agent [7]. This case reports the effectiveness of endoxifen at a dose of 8 mg OD in a young male BPAD patient with severe manic and psychotic symptoms.

## 2. Case Report

A 21-year-old young male presented to the outpatient unit at Dr. Thanvi's Neuropsychiatry and Psychotherapy Centre with complaints of reduced sleep, associated with behavioural changes like aggressiveness, excessive spending, and extravagant talking especially about opening a luxury car showroom and increased consumption of hookah/shisha smoking. He also reported that some of his cousins were conspiring with his ex-schoolmates to harm him, as they were jealous of him. Although his severity of symptoms mandated hospitalisation, his family disagreed with the same. His symptoms had persisted for more than a year. He had a strong family history of central nervous system (CNS) disorders as they were undergoing treatment at the same clinic. His mother was bearing anxiety disorder and depicted obsessive-compulsive (OC) state, his grandmother was treated at the same clinic for depression and was self-medicating with escitalopram for more than 4 years, and his aunt was also treated for anxiety disorder and OC traits.

To determine the personality characteristics, emotional functioning, and the thought process, Young Mania Rating Scale (YMRS) and Diagnostic and Statistical Manual of Mental Disorders, fifth edition (DSM-5) based psychiatric interview were performed. YMRS score was very high at 49. His differential diagnosis inferred BPAD. No physical abnormalities were present. His laboratory investigations reported slightly elevated liver enzymes, and cholesterol levels were close to the higher normal upper limit. Renal and thyroid functions were normal while glycated haemoglobin was 5.8%. The patient was receiving on and off treatment for the last more than one year under a local psychiatrist wherein he was treated with first- and second-generation antipsychotics along with mood stabilisers. His earlier treatment plan included tab. divalproate (750 mg BD), tab. risperidone+trihexyphenidyl (4 mg + 2 mg BD), tab. olanzapine (5 mg, 1 each in morning and afternoon and 2 in the night), and tab. haloperidol (5 mg TID). At our clinic, to the above prescription, tab. lithium 300 mg BD, tab. chlorpromazine (CPZ) 100 mg HS, and tab. propranolol 20 mg BD were added. Not much improvement was noted in the symptoms for the next 10 days. On the 11^th^ day, he had a YMRS score of 48, and was additionally prescribed tab. CPZ 50 mg and tab. endoxifen 8 mg both in the morning. After 8 days, there was some improvement as noted by reduced aggressive behaviour.

At the 15^th^ day, an improvement in sleep was noted, with a circadian cycle of 9 hours/day and absence of extravagant talks. At the end of the 3^rd^ week, haloperidol was gradually reduced to 5 mg HS, and risperidone was reduced to 5 mg per day. Over the next 2 weeks, olanzapine dose was reduced to 5 mg HS and CPZ to 50 mg HS. At week 8, his YMRS score reduced to 20, and his prescription now included tab. divalproate 500 mg HS, tab. lithium 300 mg BD, tab. endoxifen 8 mg in the morning, tab. risperidone 3 mg HS, and tab. propranolol 20 mg BD. Along with pharmacotherapy, psychological counselling sessions were ongoing. He was also sensitized for compliance and adherence to treatment.

**Figure 1:**
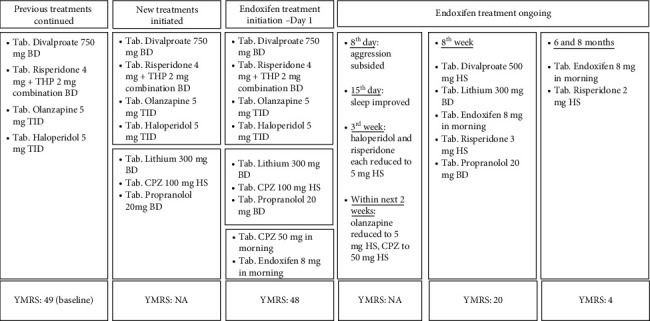
Treatment timelines.

At 6 months, the YMRS score improved to 4, which continues at 8 months. His current medications include tab. endoxifen 8 mg in morning and tab. risperidone 2 mg HS, indicating a significant reduction in his overall pill burden. During the treatment, no significant adverse events were observed to date. The patient has also reported almost nil craving for the psychostimulants—hookah/shisha smoking. The timeline of treatment is shown in Figure 1.

## 3. Discussion

The present case reports an adult male diagnosed with severe BPAD (YMRS = 49) for more than a year characterised by aggressive behaviour, grandiosity, addiction to psychostimulants, and a strong family history of psychiatric disorders. He had been on and off on first- and second-generation antipsychotics including lithium and divalproate since a year. With no improvements, endoxifen (8 mg once daily) was initiated. At day 8 of endoxifen treatment, his aggression subsided, along with sleep improvement at day 15 inferring the effectiveness of endoxifen in this patient. By the 8^th^ week, the prescription included divalproate, lithium, endoxifen, and risperidone with a YMRS score of 20. As the efficacy of allopurinol, due to its purinergic modulation in mood disorders is not well defined, it was not initiated [8, 9]. Also, as improvement with endoxifen was noted, lithium was discontinued and its blood levels were not monitored. At 6 months, observing improvements and no associated side effects (YMRS = 4), the prescription included only endoxifen and risperidone; other agents were discontinued including lithium, divalproate, and propranolol to reduce the pill burden. The improvements were maintained at the recent follow-up at 8 months.

Recent evidence implicates altered activity of intracellular PKC signalling cascade in the pathophysiology and treatment of the bipolar disorder [10]. The approved treatments for BPAD include lithium and valproate which are known indirect inhibitors of PKC, having a slower onset of action [11]. Although available treatments are effective in a substantial proportion of patients, however, 40-50% are not benefitted [6]. Numerous preclinical and clinical studies have demonstrated encouraging results of tamoxifen in mania, a relatively selective PKC inhibitor [12–14]. Endoxifen, an active metabolite of tamoxifen and a direct PKC inhibitor is fourfold potent than tamoxifen [15]. Results of a multicentre, double-blind, active-controlled phase III study using a daily dose of 8 mg endoxifen in patients with BPD I acute manic episodes with/without mixed features significantly (*p* < 0.0001) reduced the total YMRS score (from 33.1 to 17.8) and was well-tolerated for 3 weeks [15]. In the present case, although guideline-driven treatment was given for a year, the patient did not show any improvement, and thus, endoxifen at a dose of 8 mg once daily was initiated. Following initiation of endoxifen, at 8 months, the patient is tolerating the treatment well with no major adverse effects and has reported a reduction in YMRS score from 49 to 4 representing a significant improvement in his symptoms.

Recently, a case of a 52-year-old patient with severe mania reported long-term safety and effectiveness of 8 mg BID endoxifen for 3 months. During the treatment, the patient was also de-addicted from his alcohol addiction [16]. Psychostimulants, which can trigger manic episodes in susceptible persons, are known to activate PKC [17]. An *in vitro* study has reported increased PKC activity with naturally occurring cannabinoid, delta-9-tetrahydrocannabinol [18]. A critical observation in this case was that the patient's craving for hookah/shisha reduced with endoxifen treatment, which needs to be explored in further studies. Also, extensive preclinical data has demonstrated reduction in behavioral and amphetamine stimulated activities by tamoxifen and other inhibitors of PKC pathway [19, 20]. Considering cost effectiveness and the undefined relationship between serum valproate and therapeutic efficacy in affective disorders [21], the blood levels of valproic acid were not estimated. Also, other treatment regimens including clozapine were not used for the management. His regular attendance to all follow-up appointments speculated his adherence to treatment from a broader perspective [22].

## 4. Conclusion

This case demonstrates the long-term safety and efficacy of endoxifen 8 mg once daily in a BPAD patient with psychotic symptoms through improvement in YMRS score along with a reduction in the use of psychostimulants and dose of concomitant antipsychotic medications.

## Data Availability

Data are available from the authors upon reasonable request.
